# Ventricular Tachycardia from a Central Line Fracture Fragment Embolus: A Rare Complication of a Commonly Used Procedure—A Case Report and Review of the Relevant Literature

**DOI:** 10.1155/2015/265326

**Published:** 2015-12-03

**Authors:** Saptarshi Biswas, Patrick McNerney

**Affiliations:** Department of Trauma and Acute Care Surgery, Allegheny Health Network, Pittsburgh, PA, USA

## Abstract

A 22-year-old male admitted with multiple gunshot wounds (GSW) had central line placed initially for hemodynamic monitoring and later for long term antibiotics and total parenteral nutrition (TPN). On postoperative day 4 he presented with bouts of nonsustained ventricular tachycardia; the cause was unknown initially and later attributed to a catheter fragment accidentally severed and lodged in the right heart. Percutaneous retrieval technique was used to successfully extract the catheter fragment and complete recovery was achieved.

## 1. Introduction

Central catheters are widely used throughout the United States for conditions and/or treatments that require frequent intravenous access [1], to permit hemodynamic monitoring by measurement of central venous pressure, to provide long term administration of intravenous antibiotics, or to provide reliable access to provide parenteral nutrition and blood products [2].

However, despite the widespread use both catheter related infections and mechanical complications remain significantly high [3]. Complications can happen during insertion of the catheter and/or during maintenance of the line [3]. Inadvertent arterial puncture resulting in bleeding, venous thrombosis, pneumothorax, and cardiovascular side effects can all occur during insertion [4]. Central line catheter fracture/fragmentation and catheter migration are some of the rare reported mechanical complications.

We report the case of an accidental fracture of an internal jugular central line during manipulation, subsequent migration and presentation as ventricular tachyarrhythmia, and later successful retrieval by interventional percutaneous methods.

## 2. Case Report

A 20-year-old male was brought in by the EMS as an activated level 1 trauma. The patient had sustained multiple gunshot wounds to the abdomen.

On arrival the patient was alert and oriented to time and place. The patient was also complaining of abdominal pain. Primary survey revealed a patent airway, bilateral air entry on auscultation, questions answered appropriately, and movement of all his extremities. There was one wound on either side of the mid anterior abdomen as well as on the left suprapubic region and left buttock. However within a span of few minutes the patient became progressively more lethargic and obtunded. The decision was made to emergently intubate the patient and transfer to the OR. Volume resuscitation was started, with 1 liter of normal saline (NS) being given and massive transfusion activated. Initial labs showed WBC 9.19 × 10^3^/*μ*L, Hgb 10.3 g/dL, Hct 31.1%, Plt 102 × 10^3^/microliter, Na 146 mEq/L, K 4.5 mEq/L, Cl 102 mEq/L, CO_2_ 19 mEq/L, BUN 10 mg/dL, Cr 1.4 mEq/L, ALT 272 IU/L, AST 185 IU/L, ALP 51 IU/L, and TPR 0.6 ng/mL. Pre-op blood gases showed a pH of 6.8, paCO_2_ of 56 mm Hg, and HCO_3_ of 9.3 mmol/L.

**Figure 1:**
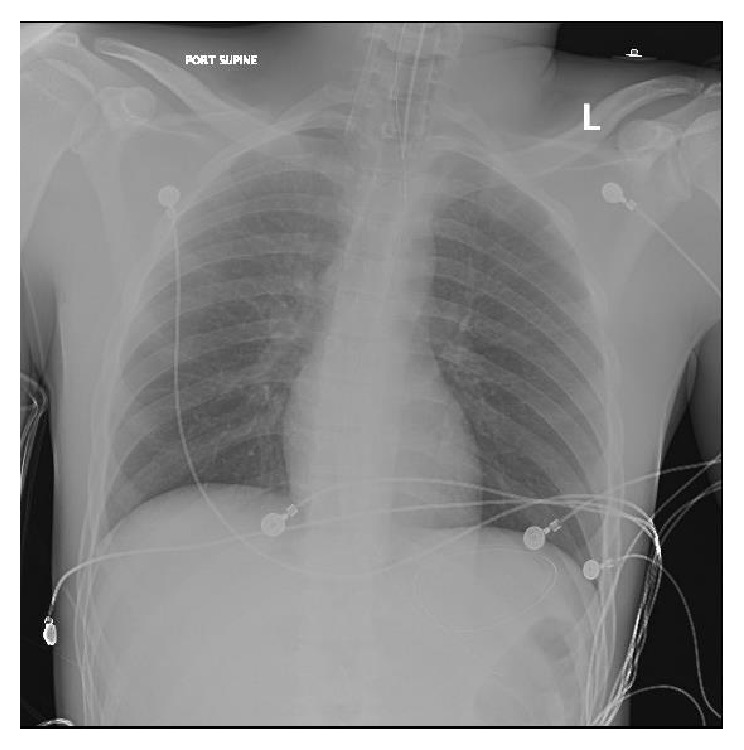
Chest X-ray post-op.

**Figure 2:**
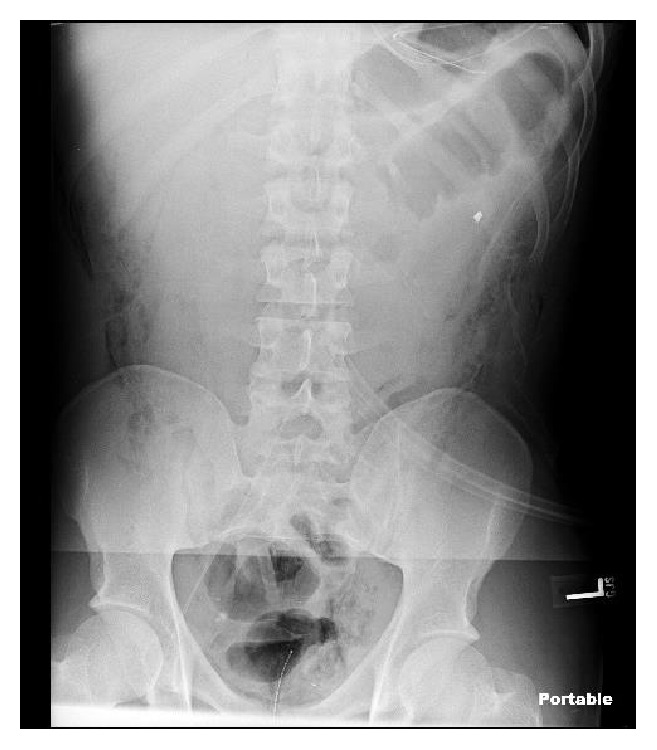
Post-op abdomen X-ray showing bullet fragment in left upper quadrant.

An exploratory laparotomy was performed emergently. Injuries to the right colic artery, 1st jejunal branch of mesentery, and 3rd portion of the duodenum were found as well as two “through and through” injuries to the small bowel and a complete transection of the descending colon that involved “fecal spillage.” The two small bowel injuries were resected and the devitalized tissue surrounding the descending colon transection was also excised. Considering the physiological status of the patient intraoperatively a damage control surgery was performed, the bowel ends were stapled, obvious bleeders were addressed, contamination was controlled, and the was abdomen packed. The abdomen was left open and covered by an Abthera wound vacuum. Post-op chest and abdominal X-rays were taken, seen in Figures 1 and 2, and the patient was transferred to the ICU for further resuscitation and stabilization of hemodynamics.

The patient was returned to the OR 3 days later. An exploratory laparotomy was performed along with one jejunojejunal anastomosis, one side to side ileocolic anastomosis, both hands sewn, closure of the mesenteric rents, and closure of the abdominal fascia. The skin was closed with staples. The patient tolerated the surgery well. The patient was reprepped and draped. A right subclavian central line was placed on second attempt after the first attempt resulted in the catheter being bunched up in the vein with brief period of arrhythmia. The patient experienced a brief period of hypotension and a chest X-ray was ordered which did not show any hemothorax or pneumothorax. The right IJ was guide wired by pulling the line back, clamping it with a hemostat and dividing it, and then going down the central lumen with a guide wire. A Mahurkar catheter was placed into the right IJ vein. Both the subclavian and the IJ dialysis lines were secured and sterile dressings applied prior to transferring the patient.

**Figure 3:**
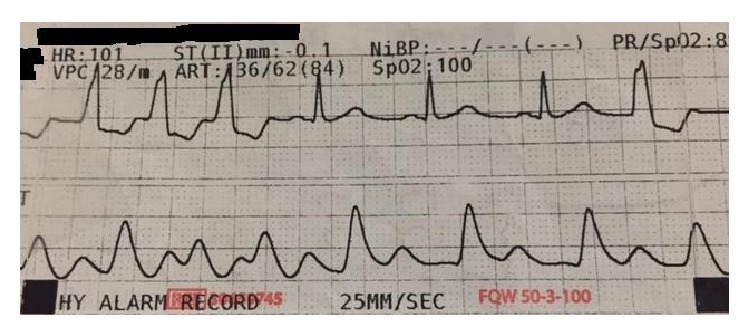
EKG strip showing ventricular tachycardia.

**Figure 4:**
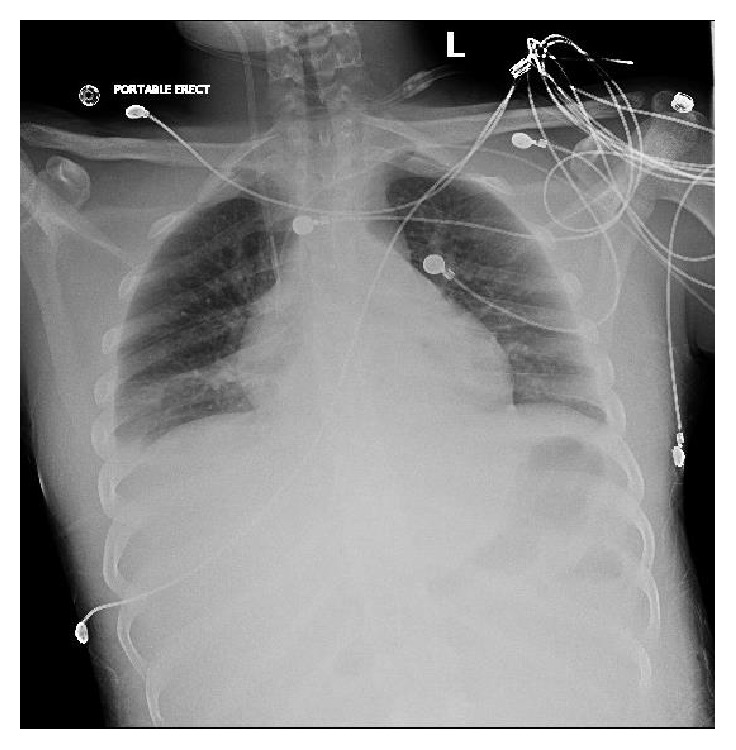
Chest X-ray AP view showing catheter projected over right atrium and superior IVC.

**Figure 5:**
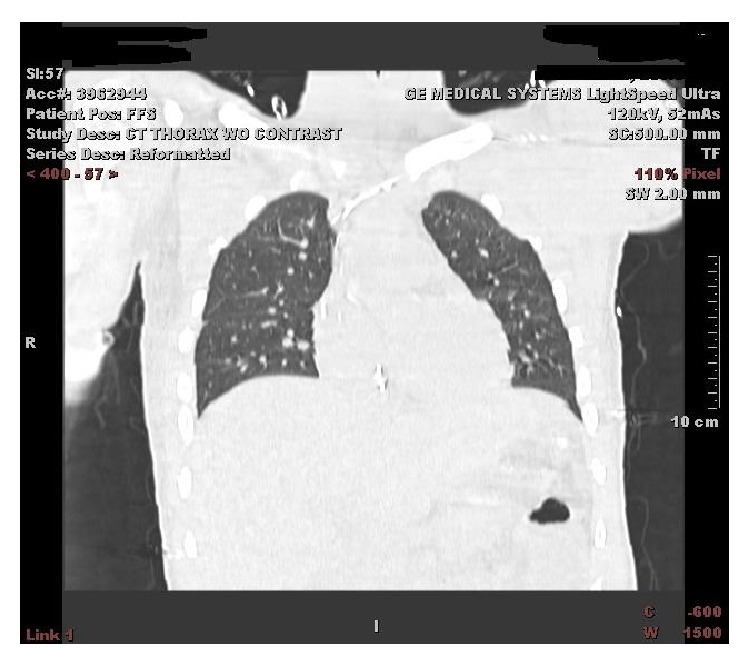
Confirmatory CT of radiopaque catheter, 10 cm, tubing extending from retrohepatic IVC through the right atrium into the low SVC.

The patient developed nonsustained runs of ventricular tachycardia 3 days after the second operation, visible in Figure 3. The EKG on admission was sinus tachycardia. Cardiology was consulted. The electrolytes were checked and with the exception of magnesium which was 1.1 mEq/L, the rest of the electrolytes were within normal range. The magnesium was replaced. The ventricular tachycardia persisted despite electrolyte replenishment and amiodarone drip was started. The amiodarone was changed to lidocaine but the patient continued to have runs of ventricular tachycardia. Four days post-op the source of the patient's arrhythmia remained a mystery until a chest X-ray revealed a piece of what was suspected to be a fractured central catheter, seen in Figure 4. The catheter fragment had lodged itself within the inferior vena cava and the right atrium. All lines and tubes connected to the patient including the EKG leads were disconnected to make sure it is not superimposed image causing confusion. A Chest CT imaging was performed which confirmed the suspicions produced by the chest X-ray, shown in Figure 5.

**Figure 6:**
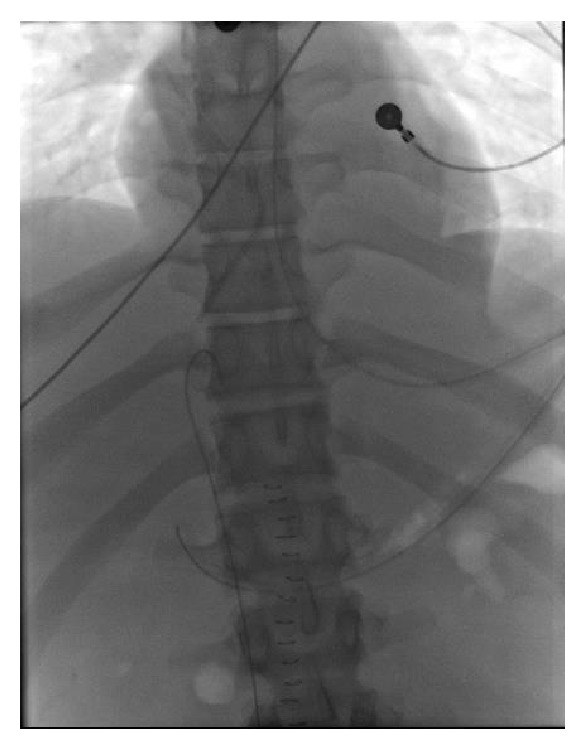
Triple loops snare used to retrieve catheter fragment.

**Figure 7:**
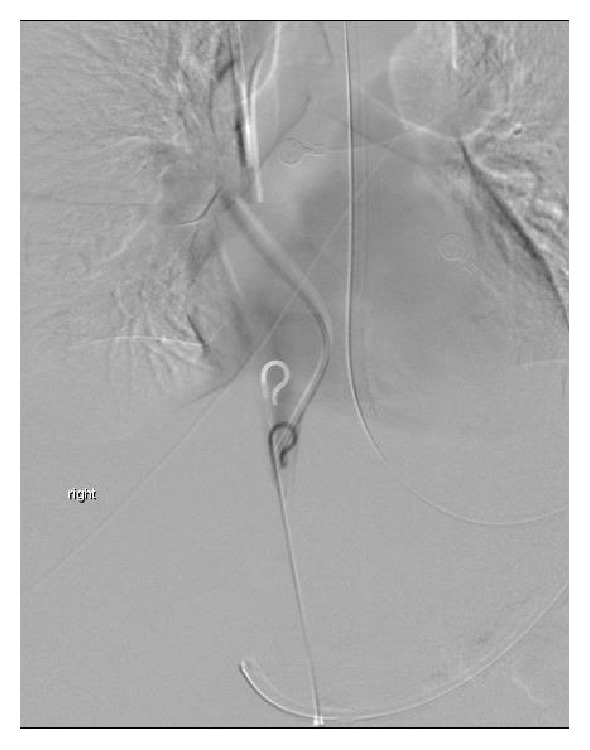
Another view of the catheter fragment retrieval procedure.

**Figure 8:**
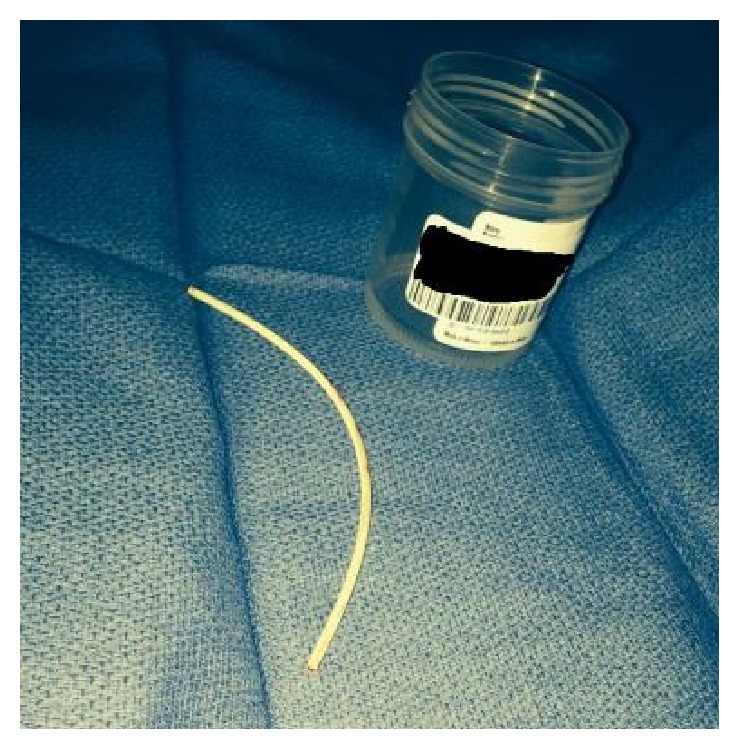
Embolized catheter fragment.

**Figure 9:**
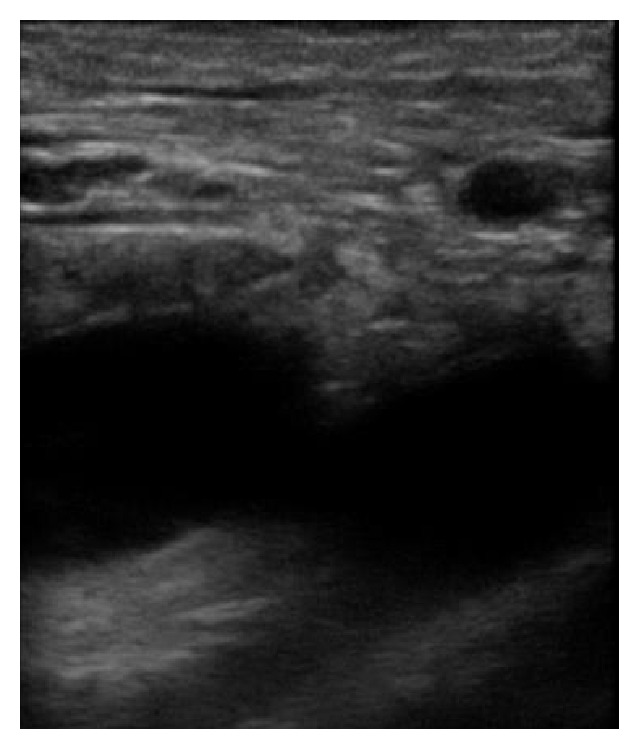
Follow-up image of IVC showing wide patency after removal of catheter fragment.

Interventional radiology was consulted and plans for immediate retrieval were made. A fluoroscopy guided percutaneous intervention resulted in retrieval of the 10 cm catheter fragment via a triple loops snare, demonstrated in Figures 6 and 7. The removed catheter fragment was briefly inspected and can be seen in Figure 8. The procedure was performed without any complications and the patient was found to tolerate it well. Repeat chest imaging confirmed successful removal of the catheter fragment, as seen in Figure 9. No further ventricular tachycardia was observed during several days of continued monitoring while the patient was in the hospital recovering from his other injuries.

## 3. Discussion

Central catheters are used in situations requiring prolonged intravenous access such as parenteral nutrition, antibiotic infusion, chemotherapy infusion, hemodialysis, or infusion of drugs known to cause phlebitis when infused directly into peripheral veins [5, 6]. Hemorrhage at the insertion site, pneumothorax, pneumohemothorax, and venous thrombosis are some of the frequent adverse events encountered from central catheter use [4]. On rare occasions severe complications like fracture/fragmentation and embolization can occur.

Surov et al. [7] had done a comprehensive review of all articles published in English literature between 1985 and 2007 [7]. He noted that Pinch-off Syndrome accounted for the majority (40.9%) and was the most common cause for catheter fragmentation [7]. Other causes sited were catheter injury during extraction (17.7%), catheter disconnection (10.7%), catheter rupture (11.6%), and unknown cause (19.1%) [7]. The catheter fracture rate was highest among central catheters inserted from peripheral veins [6]. Fracture may occur during insertion secondary to high syringe pressure or due to removal or traction on the catheter-hub junction. Loughran and Borzatta reported an incidence of 9.7% in a series of 322 applications [8]. Mortality rate was reported as 1.8% by Surov et al. in their series of 215 cases of catheter embolization [7]. The mortality depends on the duration as well as the site of embolization. Richardson et al. [9] noted that the embolized fragment lodged in the right atrium carried the highest mortality while the lowest was recorded in those in the pulmonary artery [9].

Catheter fracture has an estimated rate of occurrence of 0.1%, making it much rarer than other complications associated with central catheter use [10]. Fractured catheters have been found to have a high 71% morbidity and a 38% mortality rate [10]. Fractured catheters are reported to have travelled throughout the venous system before eventually lodging themselves somewhere. The lodged fragment can potentially obstruct blood flow or alter normal organ function. Common sites of deposition of fractured catheters are the central veins, pulmonary artery system, or within the right side of the heart [11].

Central catheter fractures can result from a multitude of situations including shearing of the catheter during insertion due to contact with the introducer needle, increased intracatheter pressure often due to bolus infusion, patient body movement resulting in fracture of the external portion of the line seen specially in infants, mechanical forces between the first rib and clavicle, and catheter fatigue due to prolonged exposure to motion of the tricuspid valve and/or right ventricle [10]. A fractured catheter can result in pulmonary embolism, cardiac arrhythmia, myocardial ischemia, valvular perforation, abscess formation, septicemia, cardiac arrest, or even sudden death and will often present with symptoms associated with those conditions [2, 5].

Catheter fatigue from use for a prolonged period of time can result in in situ fracture, as well as fragmentation and distal embolization [12]. The catheter fragment often migrates distally and finally lodges in the vena cava, right atrium, right ventricle, pulmonary artery, or its branches [10]. Interestingly the length, weight, and the material stiffness often determine the final lodgment site [10]. There have been reports of that vigorous vomiting, sneezing, or coughing has resulted in catheter tip migration [10]. Retained foreign bodies can act as a nidus for subsequent thrombus formation with resultant embolism. Endocarditis, secondary superadded infections of the thrombus, mycotic aneurysm, and pulmonary abscesses are some of the well recorded infectious complications of the process.

In some unusual situations the patient may remain asymptomatic, potentially for an extended period of time, sometimes even a number of years [4]. A case reported by Thanigaraj et al. describes a patient that was found to have a pulmonary embolism as a result of catheter fragmentation 11 years after the initial removal of the catheter [13]. Deep et al. also describe a case of an asymptomatic 80-year-old male patient, found to have an accidentally cut external portion of the central catheter with hair trimming shears, causing it to embolize [10].

Other cases have mentioned a more acute or subacute presentation. Gowda et al. report on a 34-year-old female that presented with shortness of breath and palpitations that were exacerbated when lying in the left lateral position [1]. An outpatient Holter monitor showed ventricular tachycardia that would occur when the patient was in the aforementioned position [1]. This presentation is similar to that of the patient mentioned in this case report, with the exception of this case report's patient's tachycardia not being induced by position. Chest radiograph confirmed that a catheter fragment was responsible for the symptoms after having lodged itself in the right ventricle [1]. A case described by Faircloth and Benjamin involves a less common presentation; an 8-year-old male patient that presented with shoulder pain was found via chest radiograph to have a catheter fragment embolus in his left main pulmonary artery [11]. In another male pediatric patient, aged 17, the patient complained of a cough and what he described as “feeling funny”; in this case the initial chest radiograph was incorrectly read as normal [11]. Later a chest CT was performed and the catheter fragment was identified to be present in the right atrium and ventricle after being initially described by the radiologist as a “unipolar transvenous pacemaker” [11]. Eryılmaz et al. also reported on a pediatric case; it involved a 7-year-old male that presented with a fever and signs of pneumonia [14]. A CXR was initially performed and found to be negative, a chest CT was then performed, and two catheter fragments were discovered, one in the left pulmonary artery and the other at the junction of the vena cava superior and subclavian veins [14]. These various cases allow for a general understanding of the sequela that is often present in these scenarios. Similar to a previously mentioned case, our patient suffered from a cardiac arrhythmia as a result of the catheter fragmentation, more specifically a ventricular tachycardia. The patient did not have positional arrhythmias but rather nonsustained bouts of the ventricular tachycardia that occurred while lying still and with any movement. Similar to other cases the ventricular tachycardia completely resolved on removal of the catheter fragment.

Imaging not only allows for a diagnosis of central catheter fracture but also can serve as a preventative measure. “Pinch-off Sign” is a radiological sign that appears on fluoroscopy as focal catheter narrowing that presents between the first rib and the clavicle [11]. Pinch-off Sign is a finding of “Pinch-off Syndrome” which results from catheter compression between the clavicle and the first rib, a situation that is exacerbated by excessive medial insertion of the catheter [1]. The Pinch-off Sign is one of the earliest findings associated with imminent catheter fracture [11]. Pinch-off Sign has an associated fracture risk of around 40% [11]. It has been suggested that follow-up chest radiographs spaced every 4 weeks be done on patients with central catheters as a preventive measure against catheter fracture [1]. Pain with or without swelling at the catheter site and sudden difficulty of infusion via the catheter are the first and second most common presenting signs of Pinch-off Syndrome, respectively, and are also associated with imminent catheter fracture [15].

Chest radiography and chest CT appear to be the preferred radiological modes used in the diagnosis of catheter fragmentation, with chest fluoroscopy preferred for the retrieval of the catheter fragment, if retrieval is being done via percutaneous intervention [1]. CXR and CT were used to diagnose and confirm, respectively, the catheter fragmentation found in the patient discussed in our case.

The preferred procedure for removal of a catheter fragmentation is percutaneous intervention [1]. Thomas et al. first reported in 1964 a case of nonsurgical removal of an intravascular steel guide wire fragment [16]. Percutaneous retrieval of a free floating catheter fragment has now become technique of choice. Looped wire snares, hooked guide wire, and Fogarty balloon catheters are the primary tools used in the capture of the catheter by interventional radiologists or cardiologists [10]. Percutaneous intervention retrieval of catheter fragments is generally preferred due to its relatively low adverse event rate and its greater than 95% success rate [1]. The choice of device and the technique used to retrieve the foreign body are dependent on the circumstances and the dimensions of the embolized fragment [14], since Yedlicka et al. [14, 17] gooseneck snares have gained popularity in retrieving embolized fragments. Noninvasive imaging should be done to exclude the presence of thrombus which may predispose to pulmonary embolism. Adverse events related to percutaneous retrieval of catheter fragments include blood vessel damage and/or perforation, arrhythmia, MI, stroke, insertion site bleeding, and intramural hematoma [18].

Situations do exist where percutaneous intervention is not favored or is unsuccessful, especially when both ends are fixed or entrapped and thus impossible to grasp, in which case the remaining option is surgical intervention via a thoracotomy [14]. Thoracotomy is, fortunately, rarely required with some centers reporting rates as low as 2.3% of retrievals requiring thoracotomy [14]. A triple loops snare was used to retrieve the catheter fragment via percutaneous intervention in our case, with no obvious complication.

## 4. Conclusion

Despite widespread use, central catheters are not without risk. Common risks of catheter placement include infection, hematoma, and pneumothorax [4]. Less reported complications are catheter fracture, catheter malposition, migration, cardiac perforation, and extravasation breakage [1]. Despite its rarity catheter fracture is a serious event carrying a high morbidity and mortality rate [10]. Ventricular tachycardia triggered by fragment embolism is rarely reported. Awareness of the possibility of such complication can lead to early identification and immediate management of these potentially life threatening complications, for example, septicemia, pulmonary embolism, abscess formation, arrhythmias, perforation of the great vessels or the heart, and even sudden death [13, 19].

Centrally placed catheters should be manipulated with extreme caution. Any implanted catheter should be removed after completion of the treatment and the integrity of the system should be checked on a regular basis [20]. Some authors [2] recommend early heparinization to prevent thrombus formation around catheter fragment and use of intravenous antibiotics to prevent sepsis until the time for intervention [2]. Once fractured the preferred method of retrieval is percutaneous intervention, often using a looped wire snare [10]. Thoracotomy is a less desirable method of retrieval but is at times a necessity [14].

## References

[1] Gowda M. R., Gowda R. M., Khan I. A. (2004). Positional ventricular tachycardia from a fractured mediport catheter with right ventricular migration: a case report. *Angiology*.

[2] Thapa P. B., Shrestha R., Singh D. R., Sharma S. K. (2006). Removal of central venous catheter fragment embolus in a young male. *Kathmandu University Medical Journal*.

[3] Akmal A. H., Hasan M., Mariam A. (2007). The incidence of complications of central venous catheters at an intensive care unit. *Annals of Thoracic Medicine*.

[4] Miao N.-D., Xu H., Yang L. (2014). Successful removal of a peripherally inserted central catheter fragment from the heart with a vena cava filter retrieval set. *Cardiovascular System*.

[5] Fang F., Zhang H., Yang W. (2015). An unusual peripherally inserted central catheter (PICC) fractured in vivo with embolization happened in a child: a case report. *Case Reports in Clinical Medicine*.

[6] Bashir Y., Bhat S., Manzoor F., Bashir N., Ahmad A. (2014). Catheter fracture-a rare complication of Peripherally Inserted Central Catheter (PICC). *National Journal of Medical Research*.

[7] Surov A., Wienke A., Carter J. M. (2009). Intravascular embolization of venous catheter—causes, clinical signs, and management: a systematic review. *Journal of Parenteral and Enteral Nutrition*.

[8] Loughran S. C., Borzatta M. (1995). Peripherally inserted central catheters: a report of 2506 catheter days. *Journal of Parenteral and Enteral Nutrition*.

[9] Richardson J. D., Grover F. L., Trinkle J. K. (1974). Intravenous catheter emboli: experience with twenty cases and collective review. *The American Journal of Surgery*.

[10] Deep S., Deshpande S., Howe P. (2008). Traumatic fracture of central venous catheter resulting in potential migration of distal fragment: a case report. *Cases Journal*.

[11] Faircloth J., Benjamin B. (2010). *Subclavian Central Venous Catheter Fracture and Embolization*.

[12] Dinkel H.-P., Muhm M., Exadaktylos A. K., Hoppe H., Triller J. (2002). Emergency percutaneous retrieval of a silicone port catheter fragment in pinch-off syndrome by means of an Amplatz gooseneck snare. *Emergency Radiology*.

[13] Thanigaraj S., Panneerselvam A., Yanos J. (2000). Retrieval of an IV catheter fragment from the pulmonary artery 11 years after embolization. *Chest*.

[14] Eryılmaz E., Canpolat C., Çeliker A. (2012). Catheter fragment embolization: a rare yet serious complication of catheter use in pediatric oncology. *The Turkish Journal of Pediatrics*.

[15] Cho J.-B., Park I.-Y., Sung K.-Y., Baek J.-M., Lee J.-H., Lee D.-S. (2013). Pinch-off syndrome. *Journal of the Korean Surgical Society*.

[16] Thomas J., Sinclair-Smith B., Bloomfield D., Davachi A. (1964). Non-surgical retrieval of a broken segment of steel spring guide from the right atrium and inferior vena cava. *Circulation*.

[17] Yedlicka J. W., Carlson J. E., Hunter D. W., Castañeda-Zúñiga W. R., Amplatz K. (1991). Nitinol gooseneck snare for removal of foreign bodies: experimental study and clinical evaluation. *Radiology*.

[18] Anderson H. V., Shaw R. E., Brindis R. G. (2002). A contemporary overview of percutaneous coronary interventions: the American College of Cardiology-National Cardiovascular Data Registry (ACC-NCDR). *Journal of the American College of Cardiology*.

[19] Fisher R. G., Ferreyro R. (1978). Evaluation of current techniques for nonsurgical removal of intravascular iatrogenic foreign bodies. *American Journal of Roentgenology*.

[20] Reed C. R., Sessler C. N., Glauser F. L., Phelan B. A. (1995). Central venous catheter infections: concepts and controversies. *Intensive Care Medicine*.

